# Supplementary material for: Colonizing the clinic: tracking bacterial succession and longitudinal dynamics in five new hospital departments over an entire year
Source: Microbiol Spectr. 2025 Nov 11;13(12):e02178-25. doi: 10.1128/spectrum.02178-25 (PMC12671206; doi:10.1128/spectrum.02178-25)
Supplement: Supplemental figures — Fig. S1 to S7. [file spectrum.02178-25-s0001.docx]

## Supplemental Material


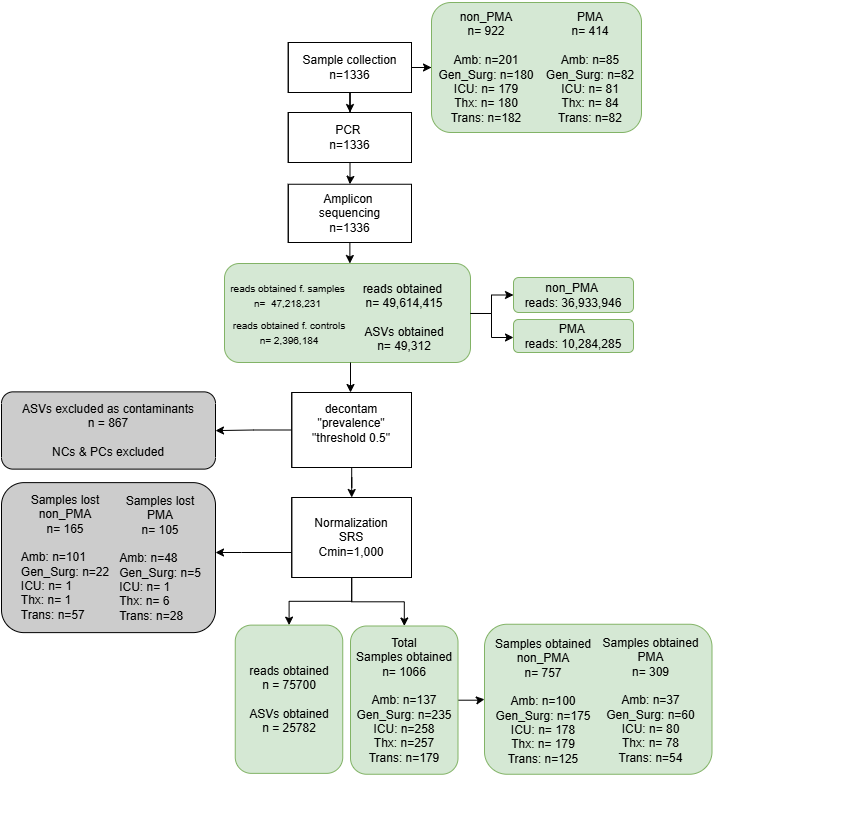


*Supplementary Figure S1: Flow chart of data acquisition and processing. Green boxes show the number of obtained samples, reads, and ASVs; grey boxes summarize excluded samples and ASVs. Abbreviations: non_PMA, untreated samples; PMA, PMA-treated samples; PMA, Propidium monoazide; Amb, Ambulatory Care Unit; Gen_Surg, General surgery; ICU, Intensive Care Unit; Thx, Thorax surgery; Trans, Transplant surgery; ASV, amplicon sequence variant.*


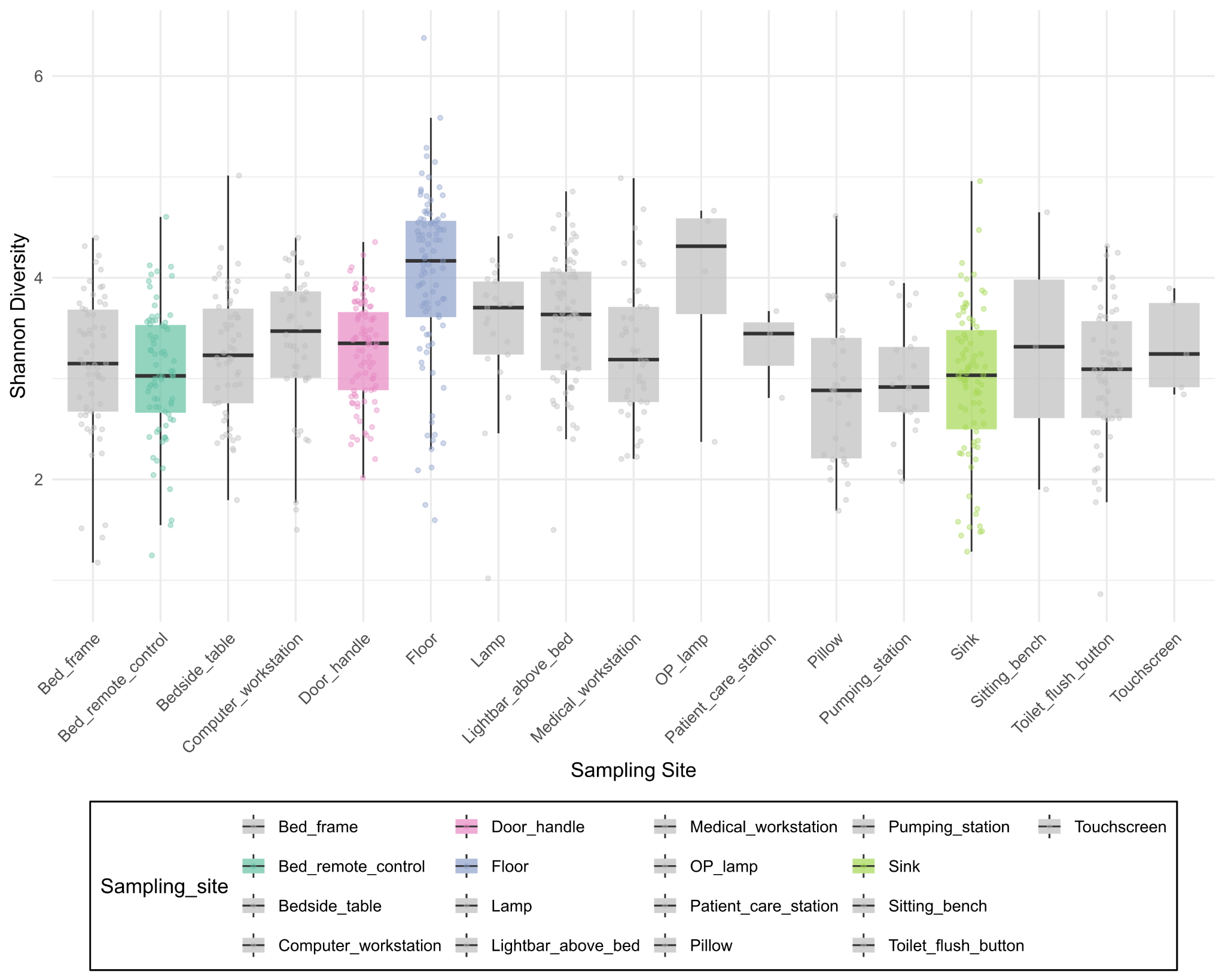


*Supplementary Figure S2: Shannon diversity at the micro-functional level. All sampling locations are shown, with the four locations sampled in all departments highlighted in color (bed remote control, door handle, floor, sink).*

##
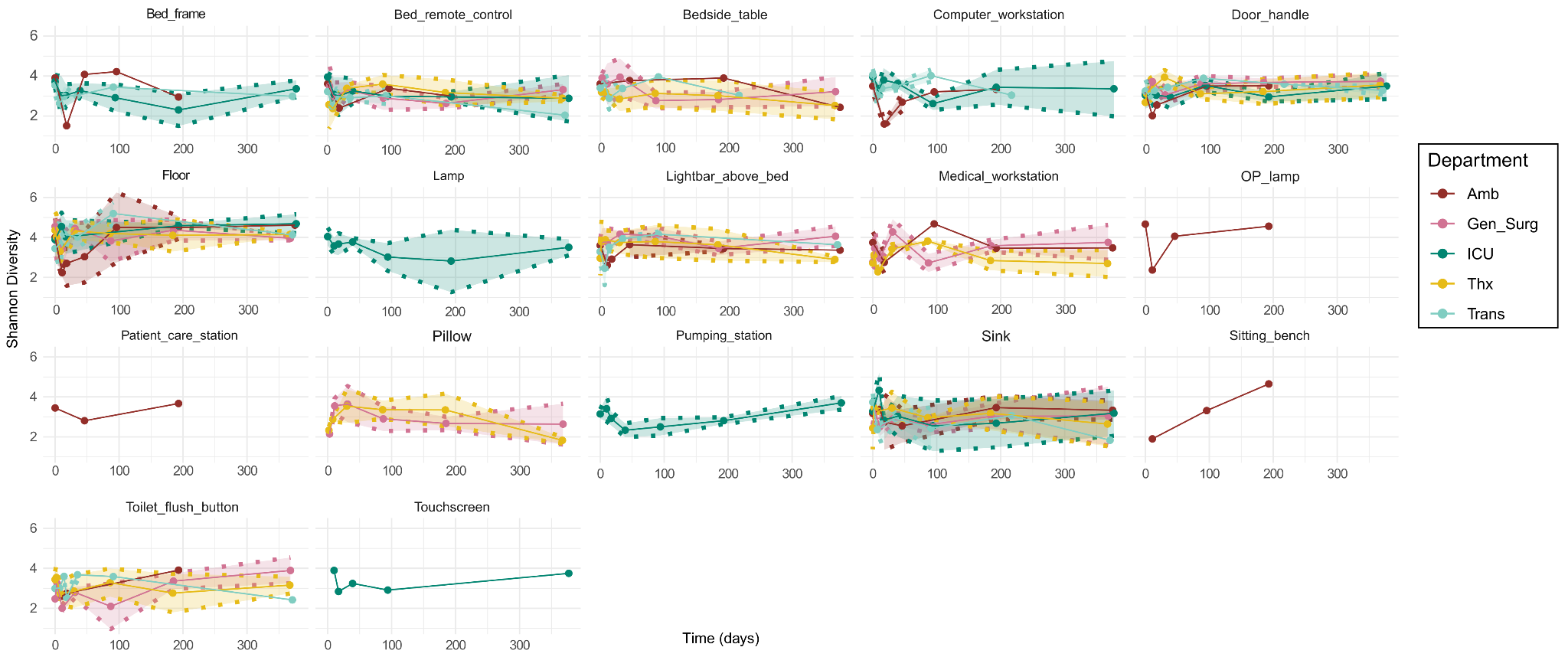


*Supplementary Figure S3: Shannon diversity at the micro-functional level over time. Shown per department.*


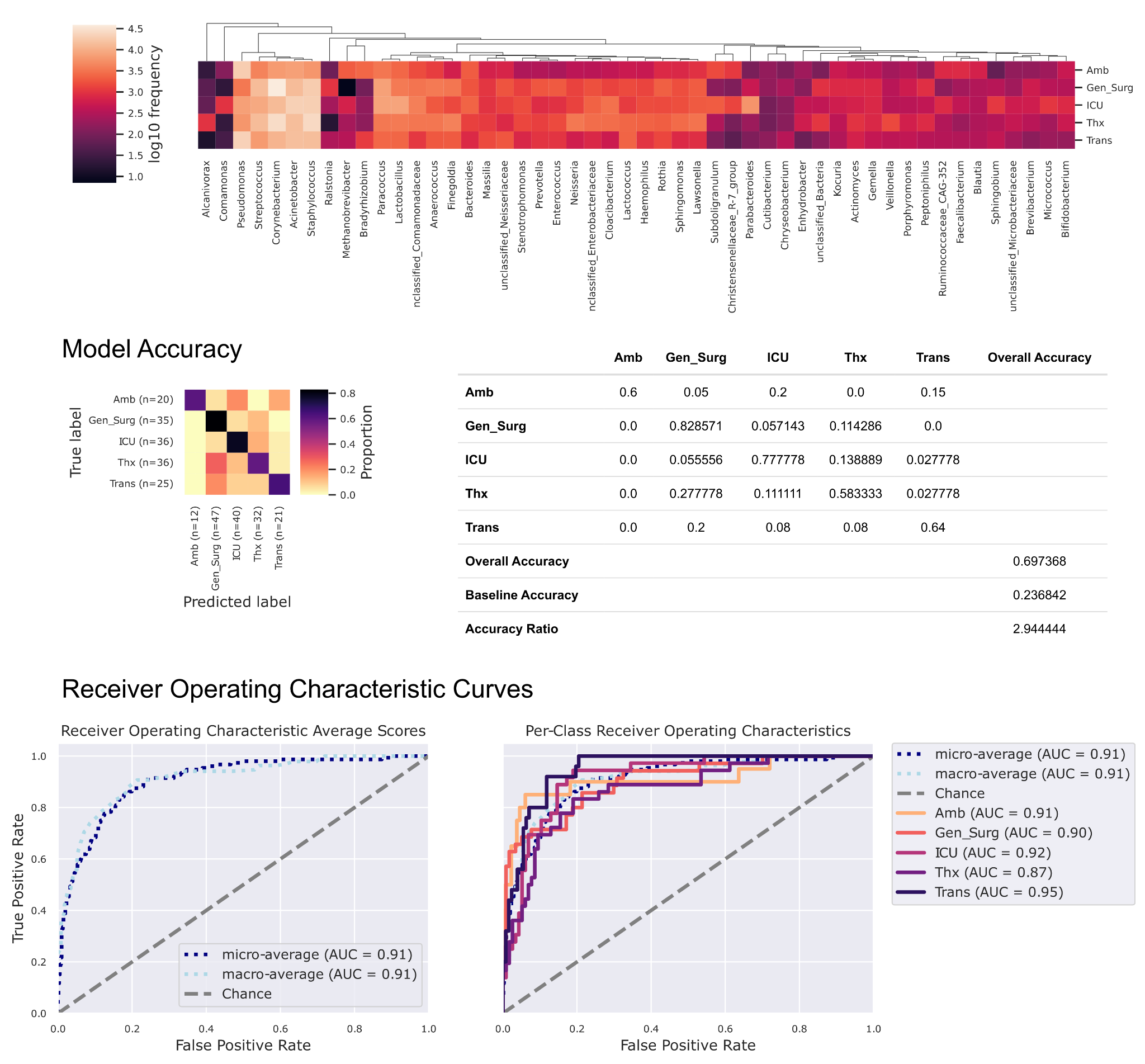


*Supplementary Figure S4: Department classification using supervised machine learning. Random Forest classifiers applied at the genus level across all time points. Model performance is shown using Receiver Operating Characteristic (ROC) curves, where the true positive rate (TPR, y-axis) is plotted against the false positive rate (FPR, x-axis). Higher area under the curve (AUC) indicates stronger classification performance; values above the diagonal grey line represent performance better than random chance.*


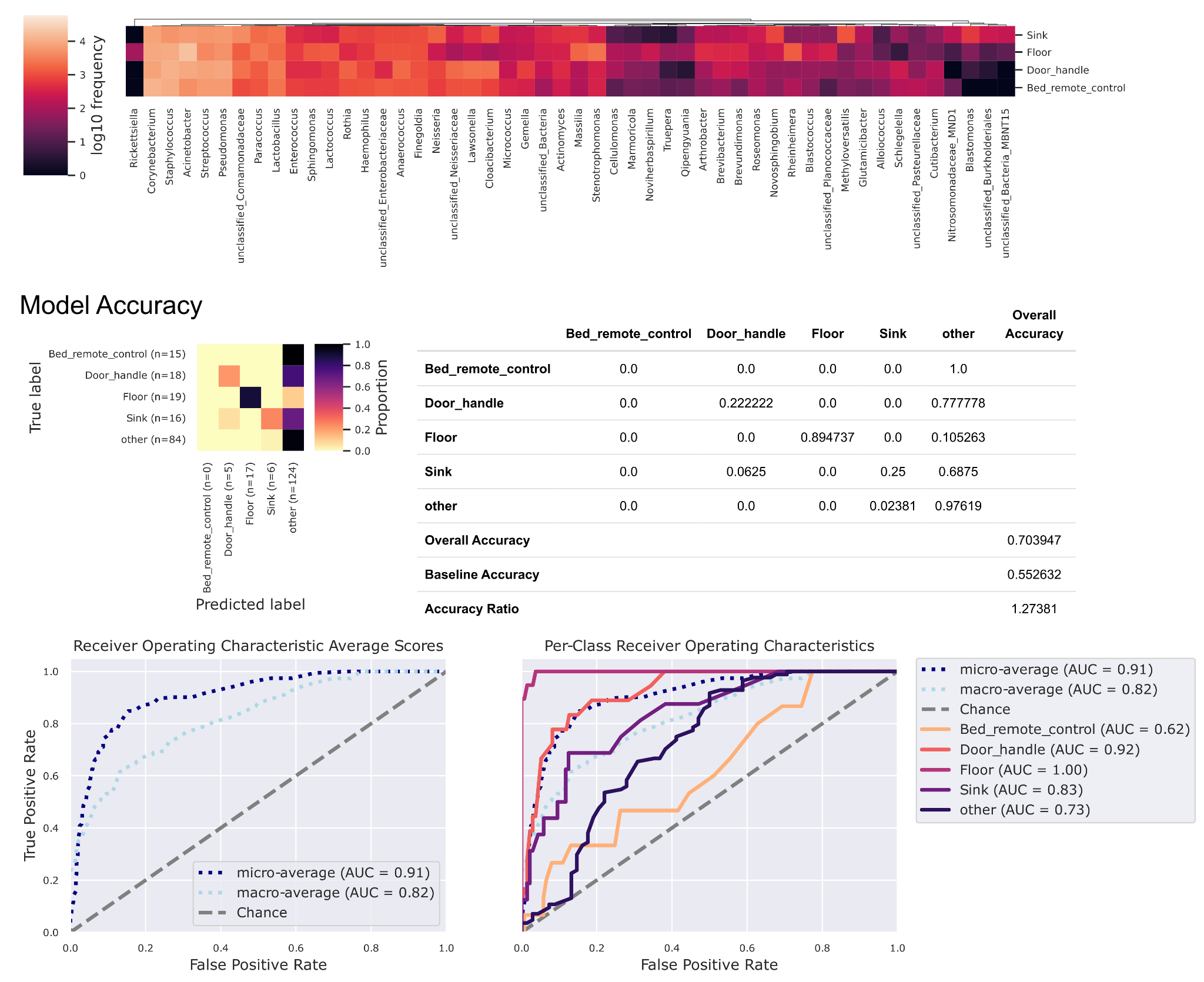


*Supplementary Figure S5: Sampling location classification using supervised machine learning. Random Forest classifiers applied at the genus level across all time points. ROC curves show the relationship between TPR (y-axis) and FPR (x-axis). Higher AUC indicates better classification, with values above the diagonal grey line representing performance above random chance.*


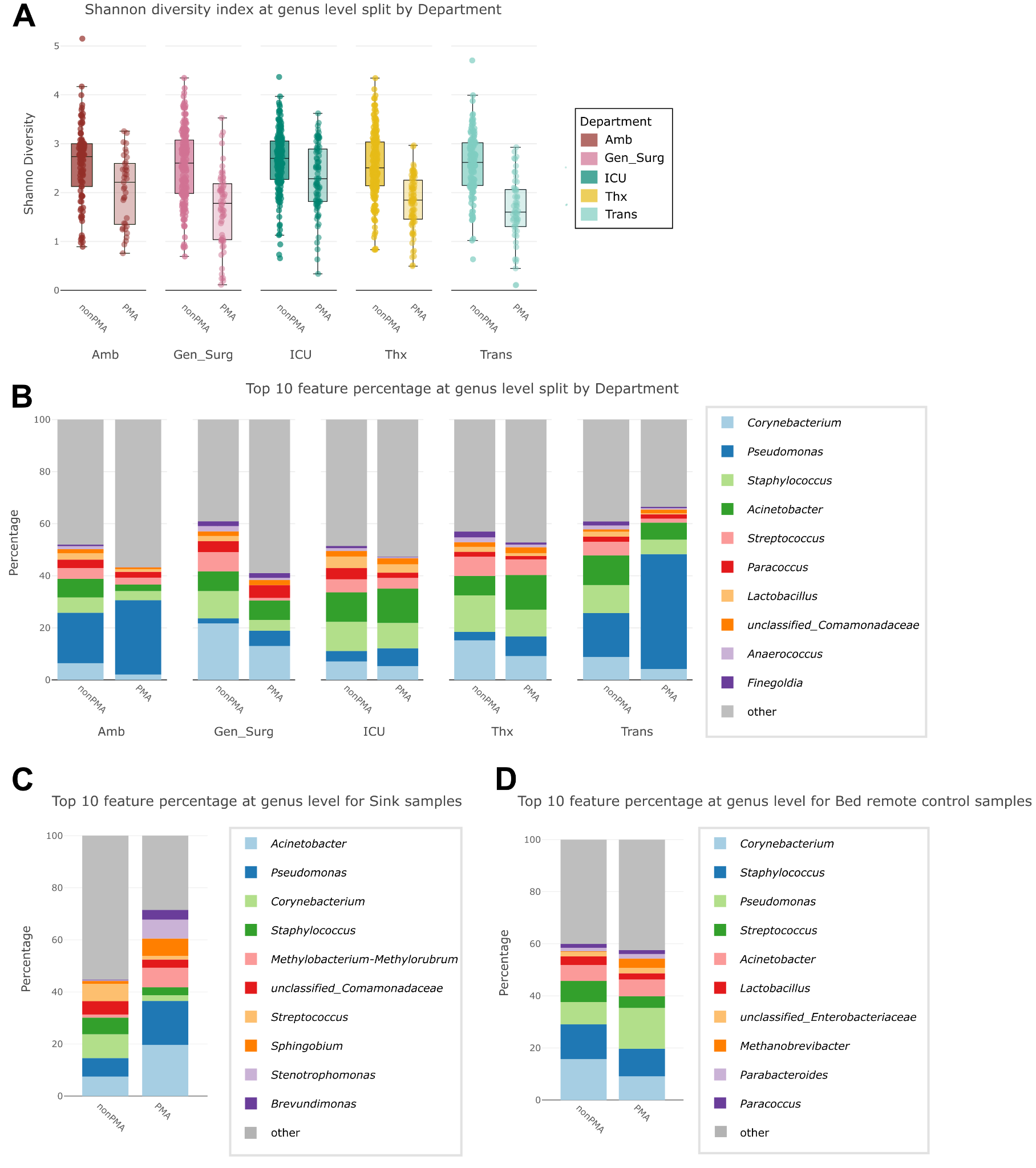


*Supplementary Figure S6: PMA treated and untreated samples. (A) Shannon diversity per department for PMA-treated and untreated (non_PMA) samples. (B) Relative abundance plots comparing PMA-treated and untreated samples. (C) Samples split by department, (D) Sink samples, and (E) bed remote control samples, treated and untreated.*


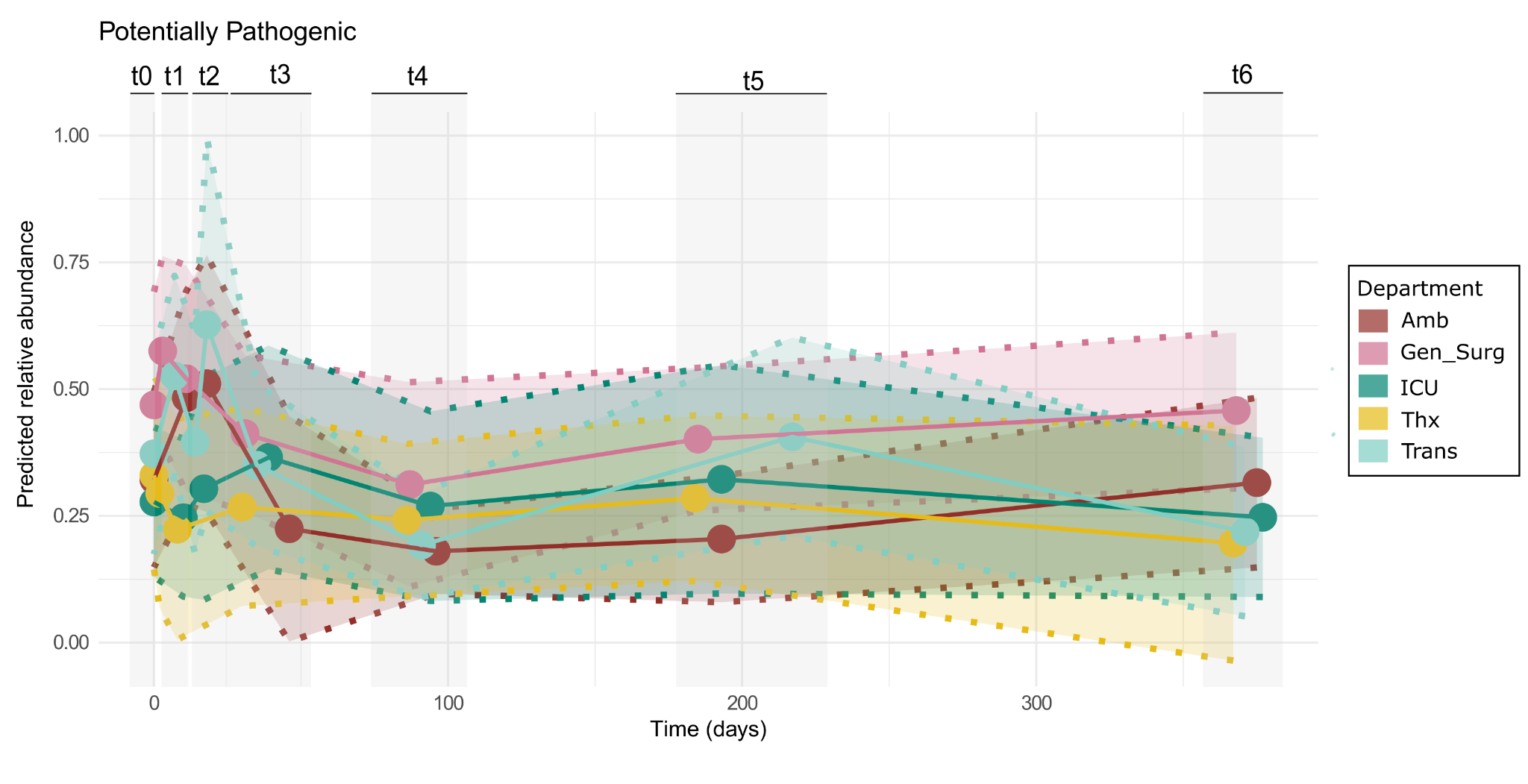


*Supplementary Figure S7: Predicted relative abundances of potential pathogenic taxa over time. Based on BugBase predictions, shown per department.*
